# Supplementary material for: A yeast phenomic model for the influence of Warburg metabolism on genetic buffering of doxorubicin
Source: Cancer Metab. 2019 Oct 23;7:9. doi: 10.1186/s40170-019-0201-3 (PMC6806529; doi:10.1186/s40170-019-0201-3)
Supplement: Supplementary file 9 — Additional file 9. GO term-specific heatmaps for REMc/GTF-enriched clusters. GO term-specific heatmaps for significant GO process terms were generated as described in methods and Figs. 3 and 4. Any related child terms are presented in subsequent pages of the parent file name. GO terms with more than 100 children, with 2 or fewer genes annotated to the term, or a file size over 300KB are not shown. All heatmaps are generated with the same layout (see Figs. 3 and 4). [file 40170_2019_201_MOESM9_ESM.bz2 › Additional_File9_GOTermHeatmaps/Additional_File9_GOTermHeatmap/ribonucleotide_biosynthetic_process.pdf]

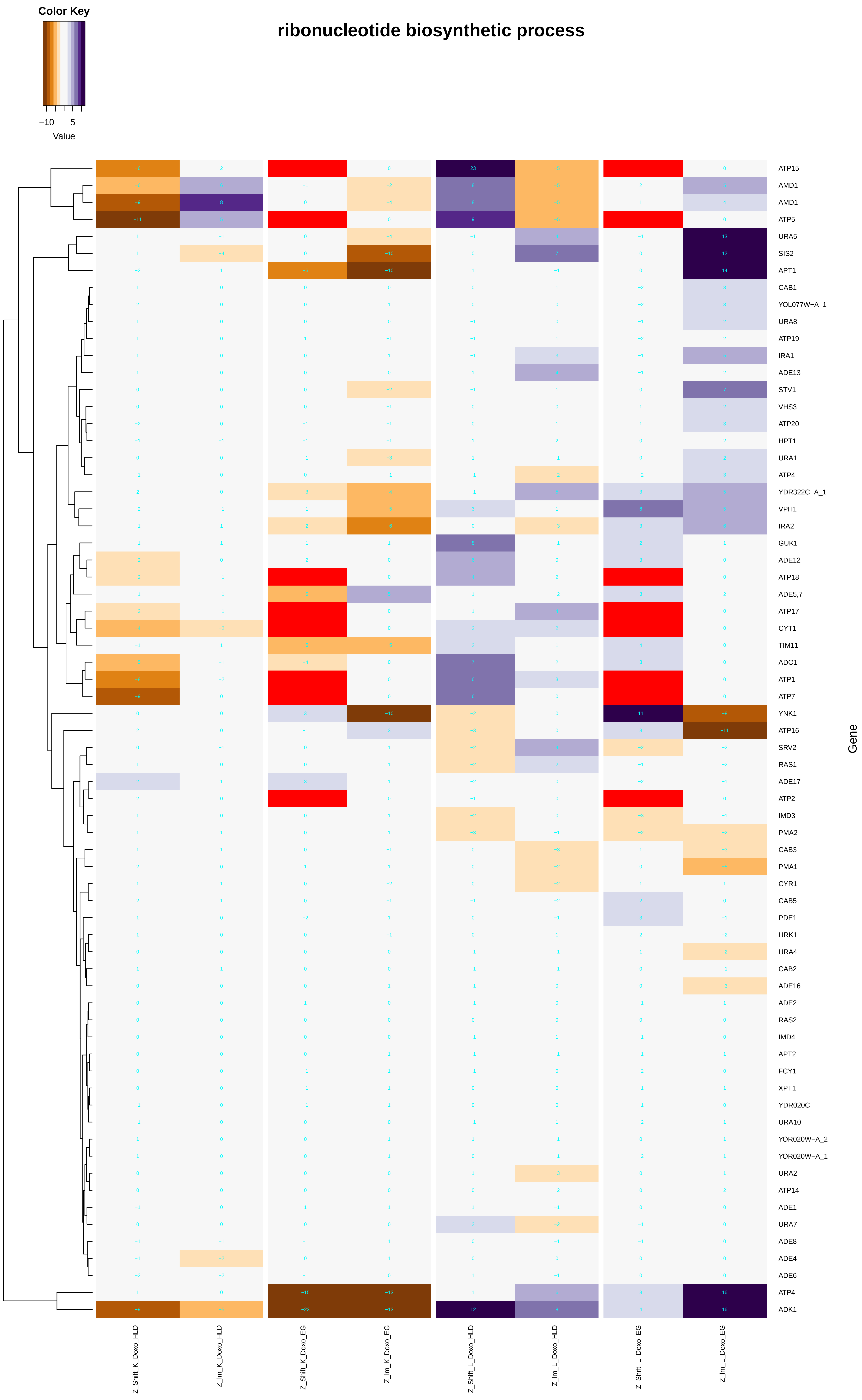
Color Key
−10
5
Value
ribonucleotide biosynthetic process
ATP15
AMD1
AMD1
ATP5
URA5
SIS2
APT1
CAB1
YOL077W−A_1
URA8
ATP19
IRA1
ADE13
STV1
VHS3
ATP20
HPT1
URA1
ATP4
YDR322C−A_1
VPH1
IRA2
GUK1
ADE12
ATP18
ADE5,7
ATP17
CYT1
TIM11
ADO1
ATP1
ATP7
YNK1
ATP16
SRV2
RAS1
ADE17
ATP2
IMD3
PMA2
CAB3
PMA1
CYR1
CAB5
PDE1
URK1
URA4
CAB2
ADE16
ADE2
RAS2
IMD4
APT2
FCY1
XPT1
YDR020C
URA10
YOR020W−A_2
YOR020W−A_1
URA2
ATP14
ADE1
URA7
ADE8
ADE4
ADE6
ATP4
ADK1
Gene
Z_Shift_K_Doxo_HLD
Z_Im_K_Doxo_HLD
Z_Shift_K_Doxo_EG
Z_Im_K_Doxo_EG
Z_Shift_L_Doxo_HLD
Z_Im_L_Doxo_HLD
Z_Shift_L_Doxo_EG
Z_Im_L_Doxo_EG

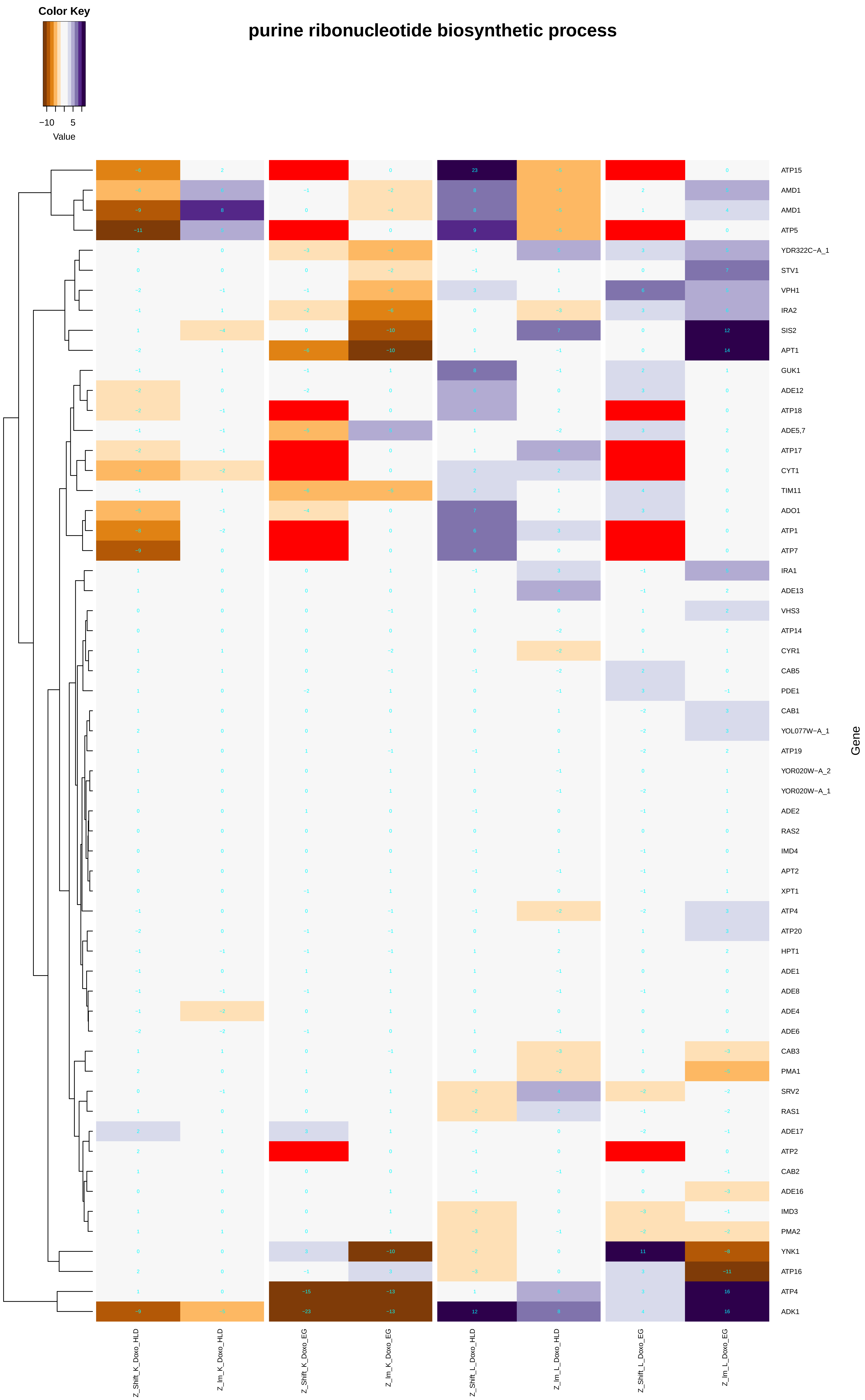

Color Key
−10
5
Value
purine ribonucleotide biosynthetic process
ATP15
AMD1
AMD1
ATP5
YDR322C−A_1
STV1
VPH1
IRA2
SIS2
APT1
GUK1
ADE12
ATP18
ADE5,7
ATP17
CYT1
TIM11
ADO1
ATP1
ATP7
IRA1
ADE13
VHS3
ATP14
CYR1
CAB5
PDE1
CAB1
YOL077W−A_1
ATP19
YOR020W−A_2
YOR020W−A_1
ADE2
RAS2
IMD4
APT2
XPT1
ATP4
ATP20
HPT1
ADE1
ADE8
ADE4
ADE6
CAB3
PMA1
SRV2
RAS1
ADE17
ATP2
CAB2
ADE16
IMD3
PMA2
YNK1
ATP16
ATP4
ADK1
Gene
Z_Shift_K_Doxo_HLD
Z_Im_K_Doxo_HLD
Z_Shift_K_Doxo_EG
Z_Im_K_Doxo_EG
Z_Shift_L_Doxo_HLD
Z_Im_L_Doxo_HLD
Z_Shift_L_Doxo_EG
Z_Im_L_Doxo_EG

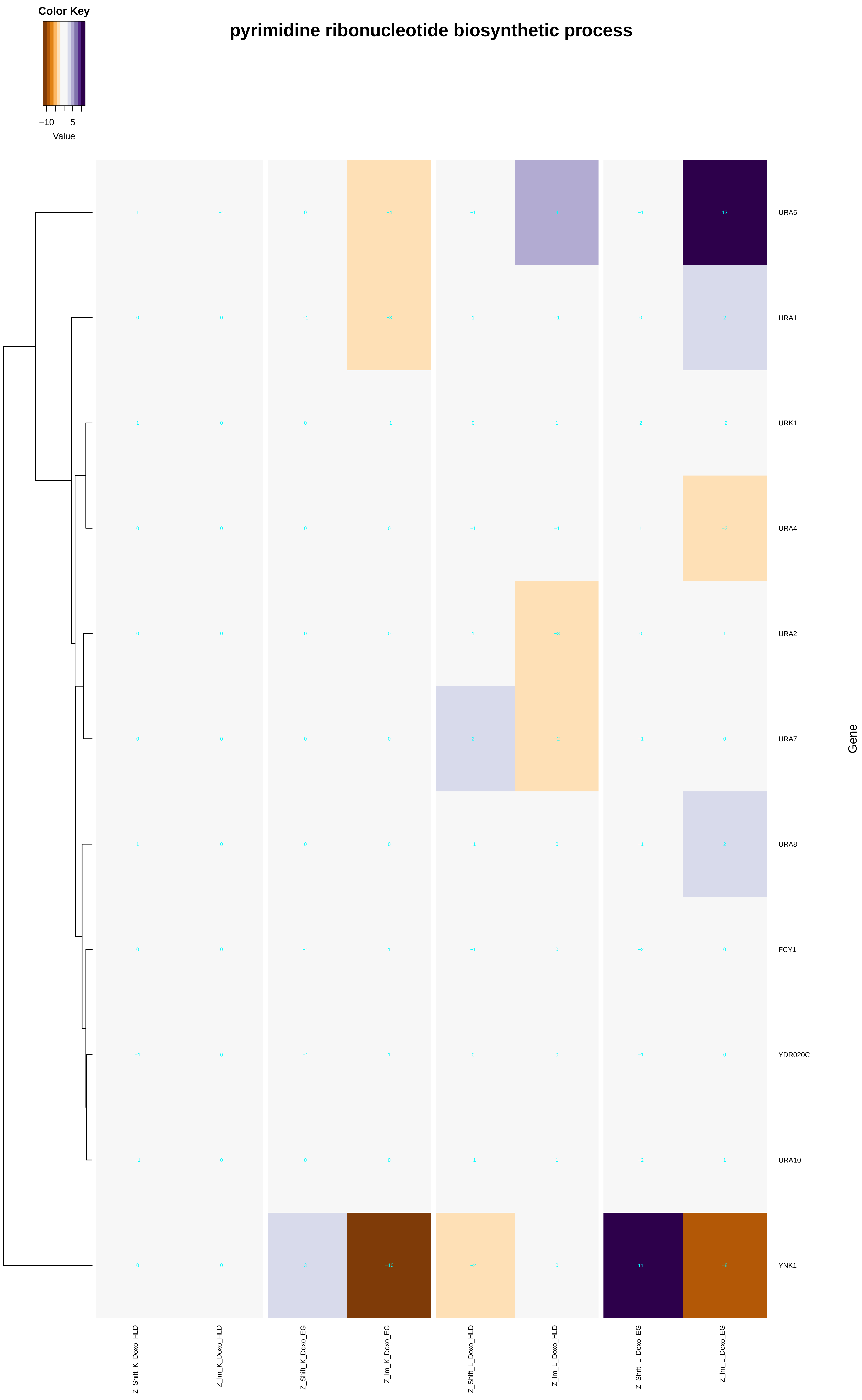
Color Key
−10
5
Value
pyrimidine ribonucleotide biosynthetic process
URA5
URA1
URK1
URA4
URA2
URA7
URA8
FCY1
YDR020C
URA10
YNK1
Gene
Z_Shift_K_Doxo_HLD
Z_Im_K_Doxo_HLD
Z_Shift_K_Doxo_EG
Z_Im_K_Doxo_EG
Z_Shift_L_Doxo_HLD
Z_Im_L_Doxo_HLD
Z_Shift_L_Doxo_EG
Z_Im_L_Doxo_EG

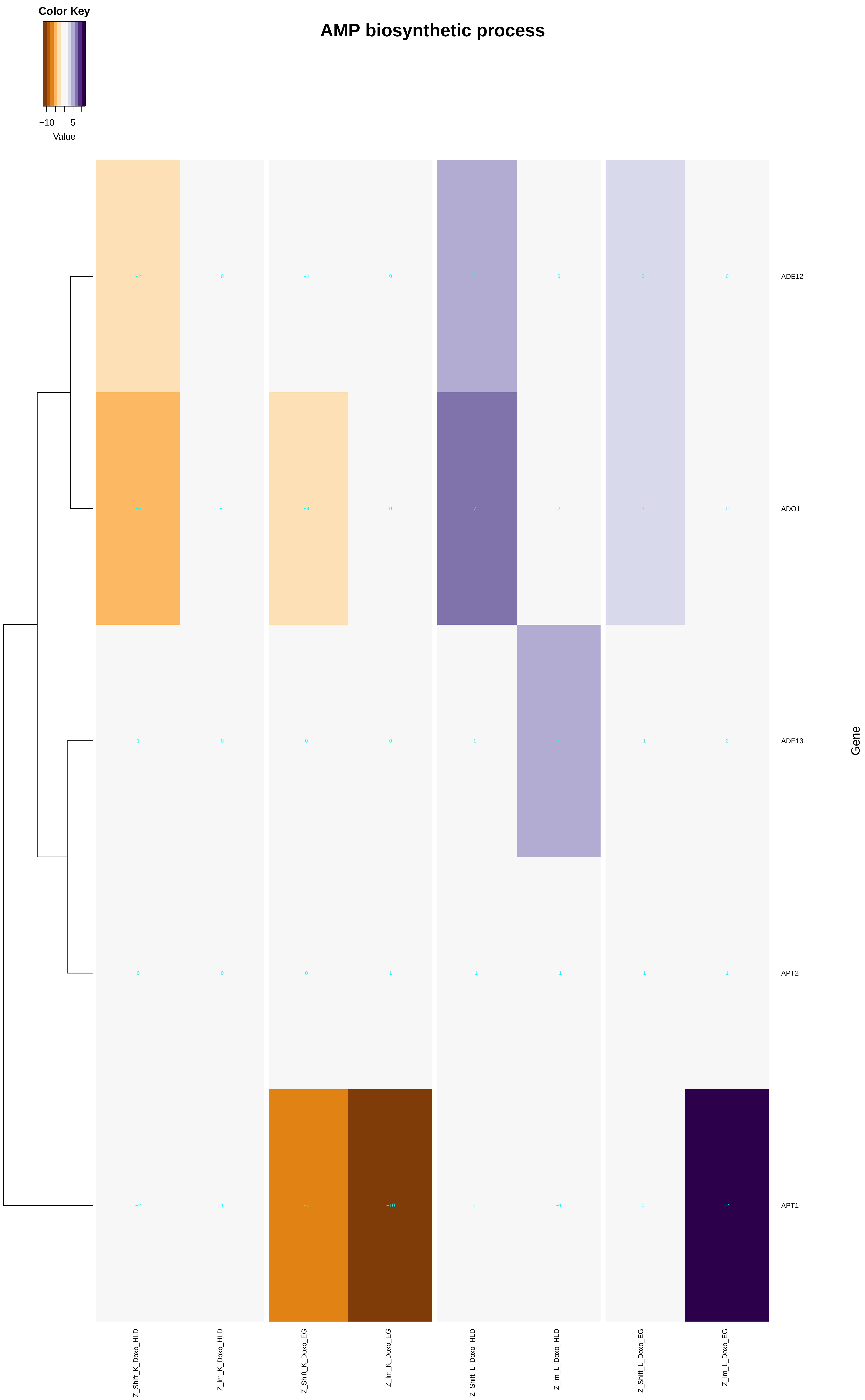
AMP biosynthetic process
Color Key
−10
5
Value
ADE12
ADO1
ADE13
APT2
APT1
Gene
Z_Shift_K_Doxo_HLD
Z_Im_K_Doxo_HLD
Z_Shift_K_Doxo_EG
Z_Im_K_Doxo_EG
Z_Shift_L_Doxo_HLD
Z_Im_L_Doxo_HLD
Z_Shift_L_Doxo_EG
Z_Im_L_Doxo_EG

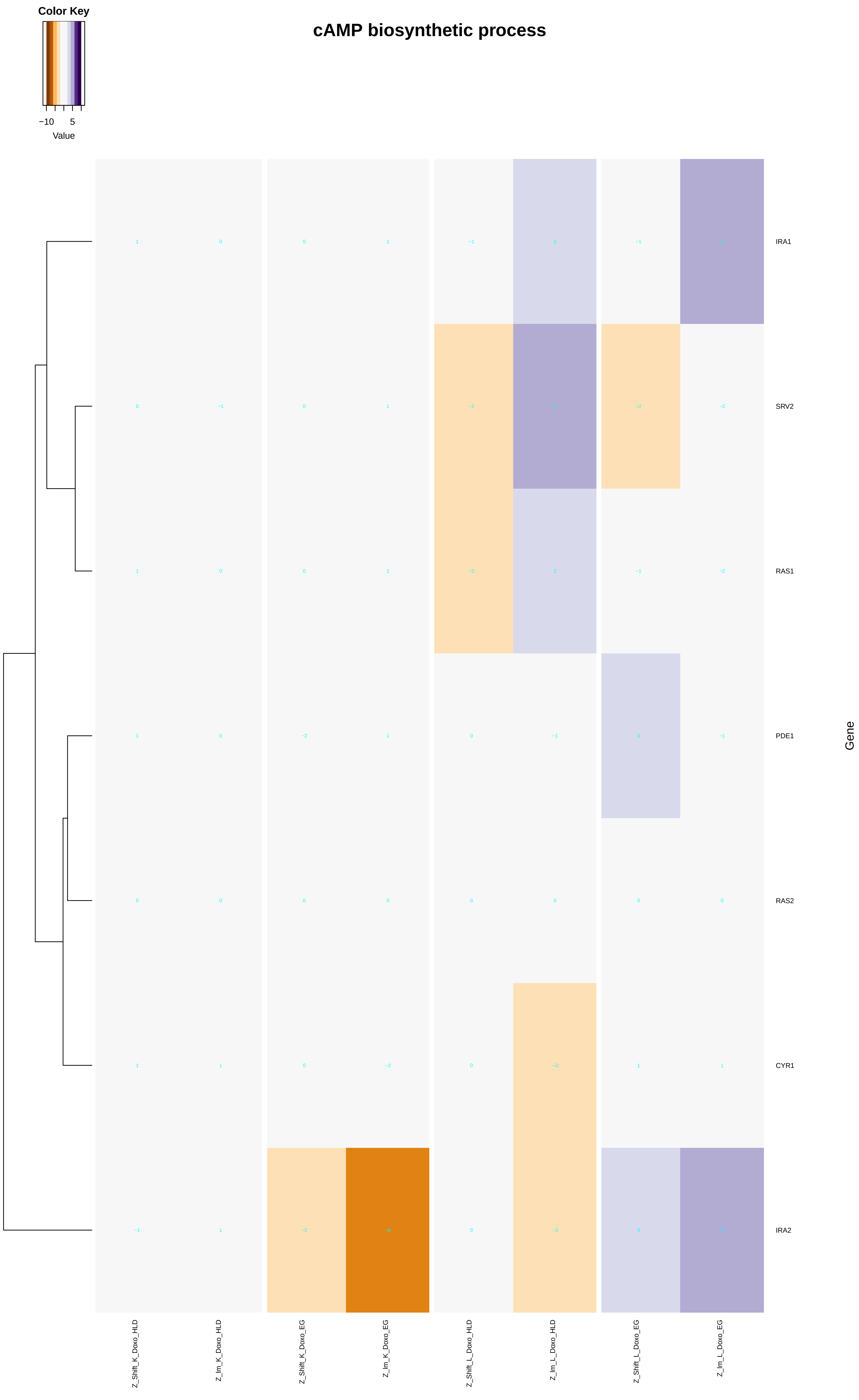

Color Key
−10
5
Value
cAMP biosynthetic process
IRA1
SRV2
RAS1
PDE1
RAS2
CYR1
IRA2
Gene
Z_Shift_K_Doxo_HLD
Z_Im_K_Doxo_HLD
Z_Shift_K_Doxo_EG
Z_Im_K_Doxo_EG
Z_Shift_L_Doxo_HLD
Z_Im_L_Doxo_HLD
Z_Shift_L_Doxo_EG
Z_Im_L_Doxo_EG

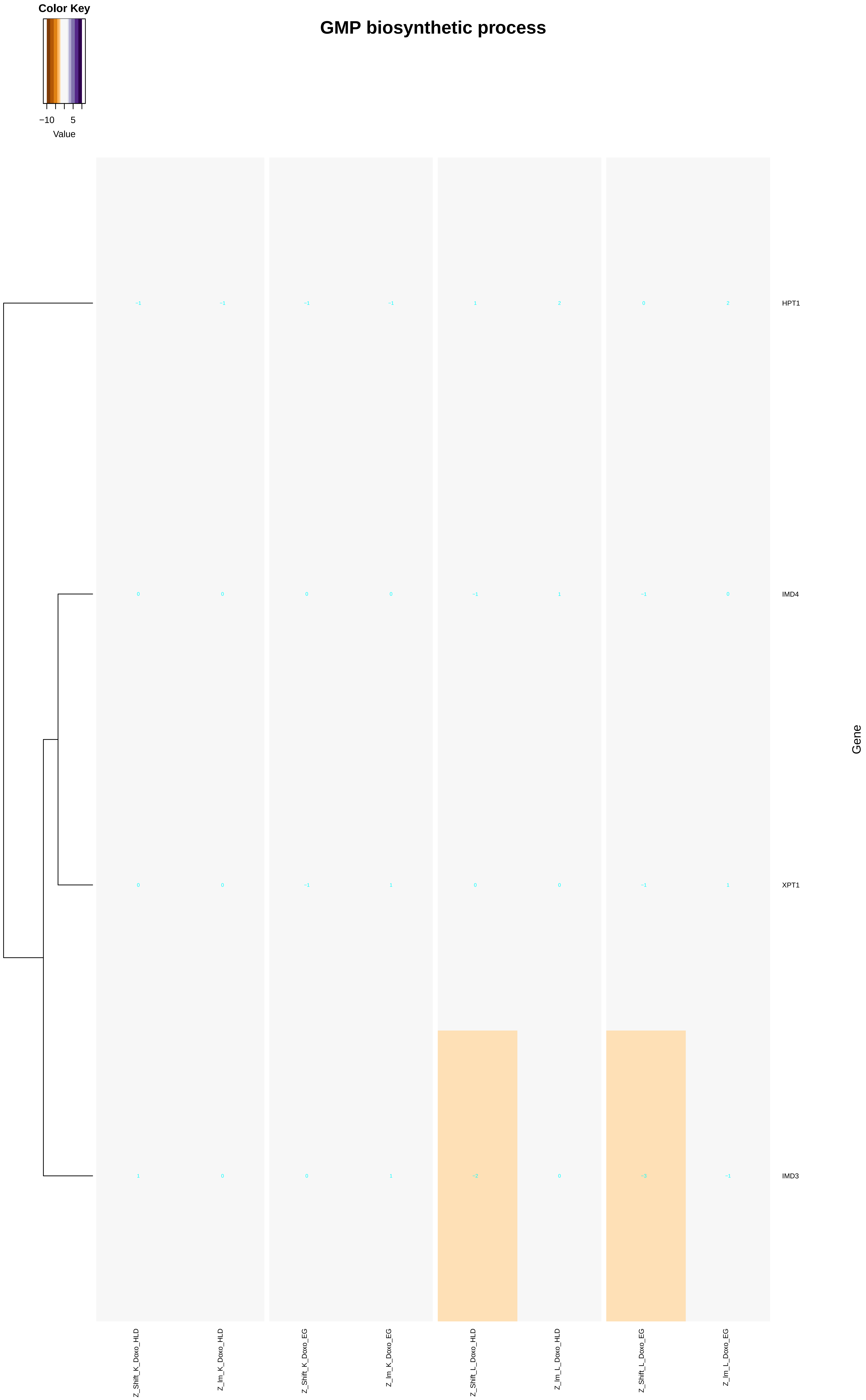

GMP biosynthetic process
Color Key
−10
5
Value
HPT1
IMD4
XPT1
IMD3
Gene
Z_Shift_K_Doxo_HLD
Z_lm_K_Doxo_HLD
Z_Shift_K_Doxo_EG
Z_lm_K_Doxo_EG
Z_Shift_L_Doxo_HLD
Z_lm_L_Doxo_HLD
Z_Shift_L_Doxo_EG
Z_lm_L_Doxo_EG

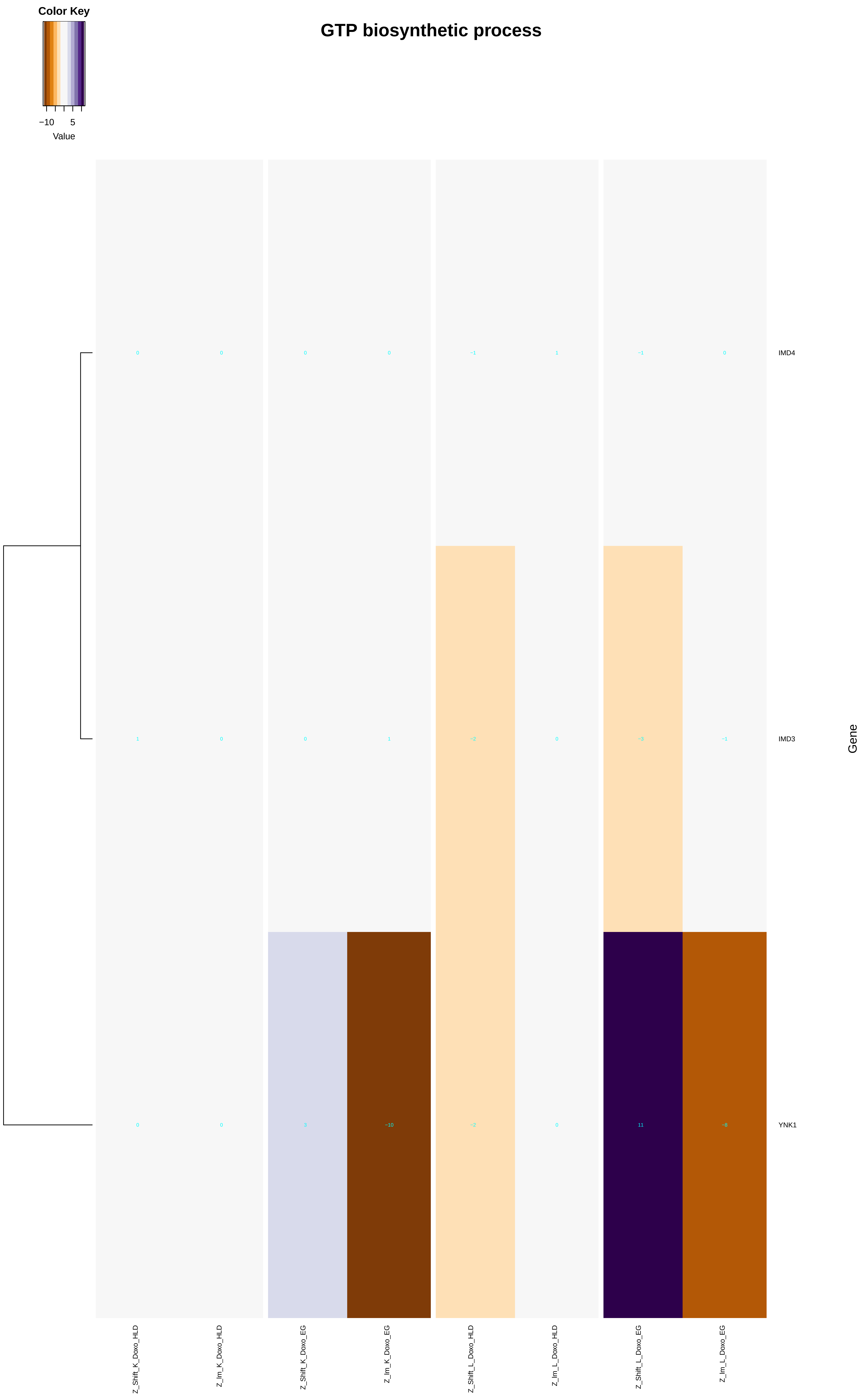
Color Key
−10
5
Value
GTP biosynthetic process
IMD4
IMD3
YNK1
Gene
0 0 0 0 −1 1 −1 0
1 0 0 1 −2 0 −3 −1
0 0 3 −10 −2 0 11 −8
Z_Shift_K_Doxo_HLD
Z_Im_K_Doxo_HLD
Z_Shift_K_Doxo_EG
Z_Im_K_Doxo_EG
Z_Shift_L_Doxo_HLD
Z_Im_L_Doxo_HLD
Z_Shift_L_Doxo_EG
Z_Im_L_Doxo_EG

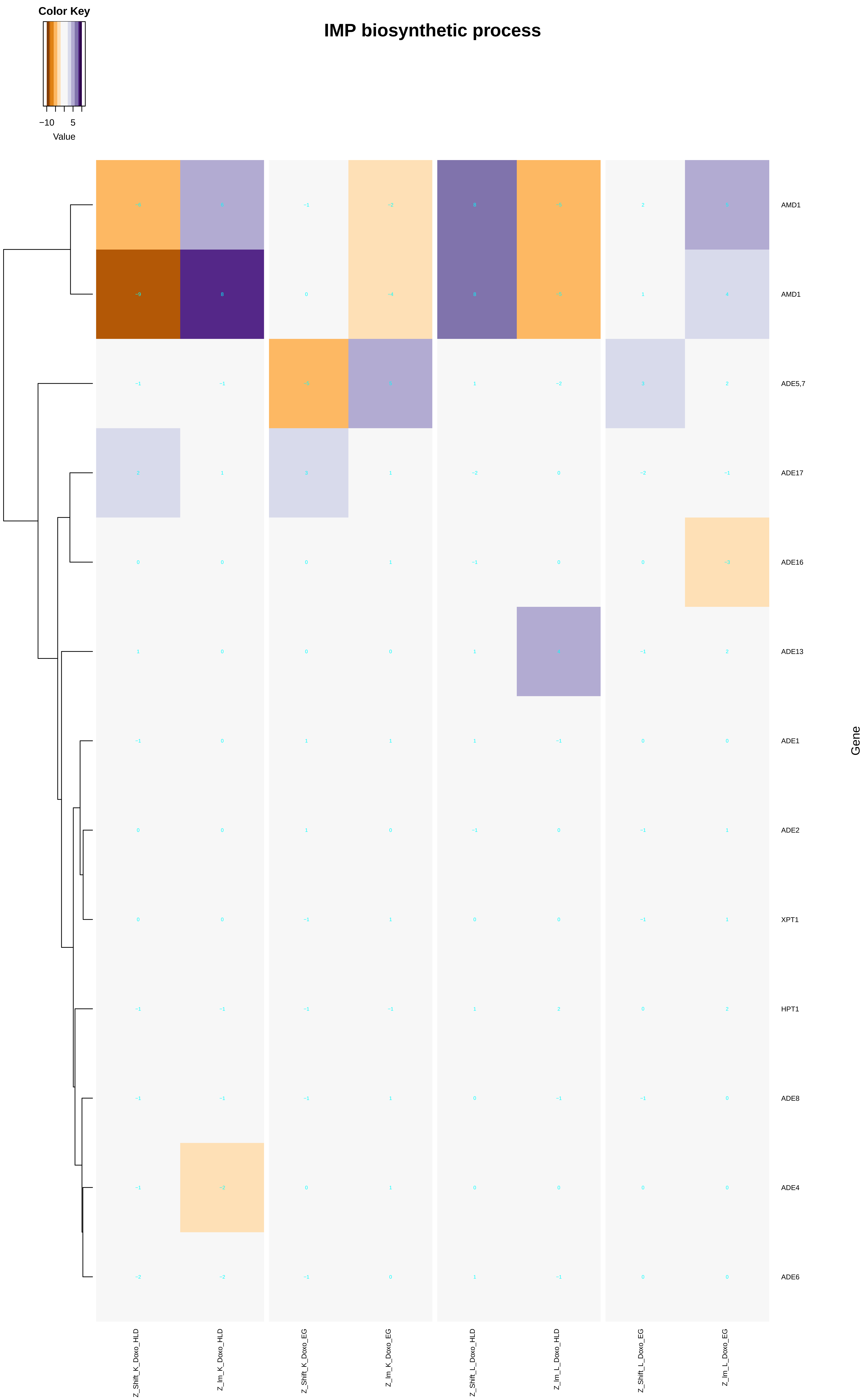
IMP biosynthetic process
Color Key
−10
5
Value
AMD1
AMD1
ADE5,7
ADE17
ADE16
ADE13
ADE1
ADE2
XPT1
HPT1
ADE8
ADE4
ADE6
Gene
Z_Shift_K_Doxo_HLD
Z_Im_K_Doxo_HLD
Z_Shift_K_Doxo_EG
Z_Im_K_Doxo_EG
Z_Shift_L_Doxo_HLD
Z_Im_L_Doxo_HLD
Z_Shift_L_Doxo_EG
Z_Im_L_Doxo_EG

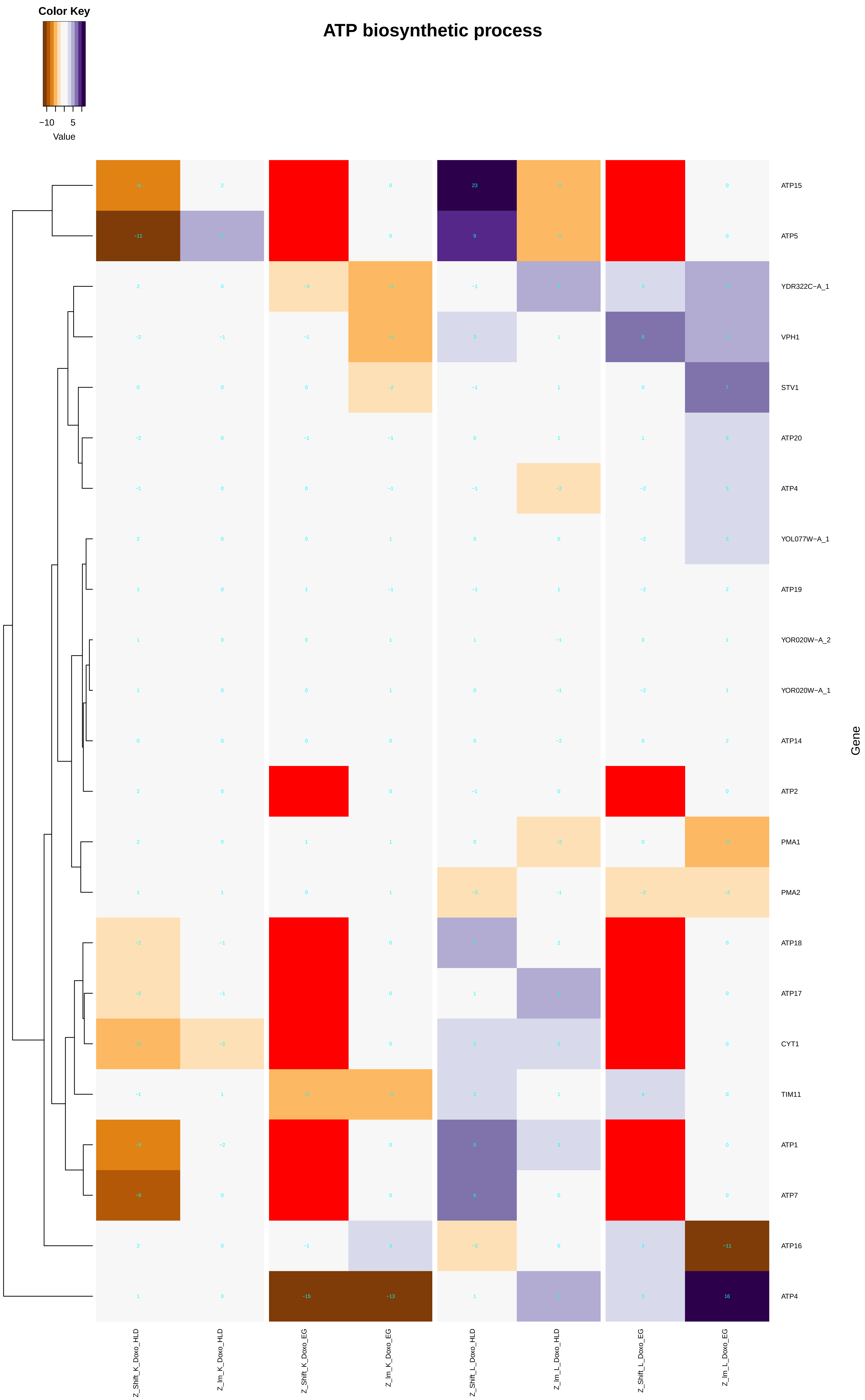
ATP biosynthetic process
Color Key
−10
5
Value
ATP15
ATP5
YDR322C−A_1
VPH1
STV1
ATP20
ATP4
YOL077W−A_1
ATP19
YOR020W−A_2
YOR020W−A_1
ATP14
ATP2
PMA1
PMA2
ATP18
ATP17
CYT1
TIM11
ATP1
ATP7
ATP16
ATP4
Gene
Z_Shift_K_Doxo_HLD
Z_lm_K_Doxo_HLD
Z_Shift_K_Doxo_EG
Z_lm_K_Doxo_EG
Z_Shift_L_Doxo_HLD
Z_lm_L_Doxo_HLD
Z_Shift_L_Doxo_EG
Z_lm_L_Doxo_EG

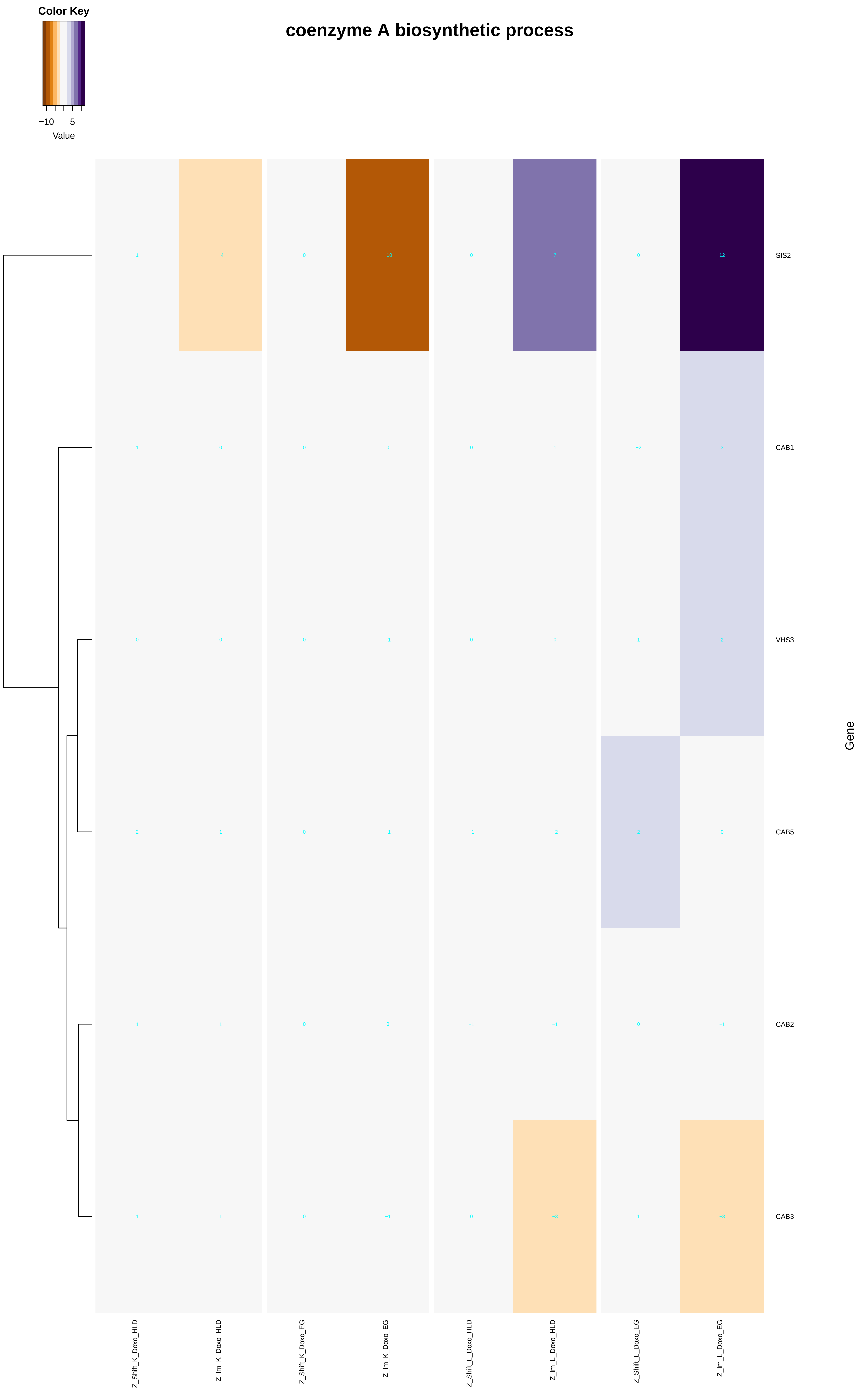
Color Key
−10
5
Value
coenzyme A biosynthetic process
SIS2
CAB1
VHS3
CAB5
CAB2
CAB3
Gene
Z_Shift_K_Doxo_HLD
Z_Im_K_Doxo_HLD
Z_Shift_K_Doxo_EG
Z_Im_K_Doxo_EG
Z_Shift_L_Doxo_HLD
Z_Im_L_Doxo_HLD
Z_Shift_L_Doxo_EG
Z_Im_L_Doxo_EG

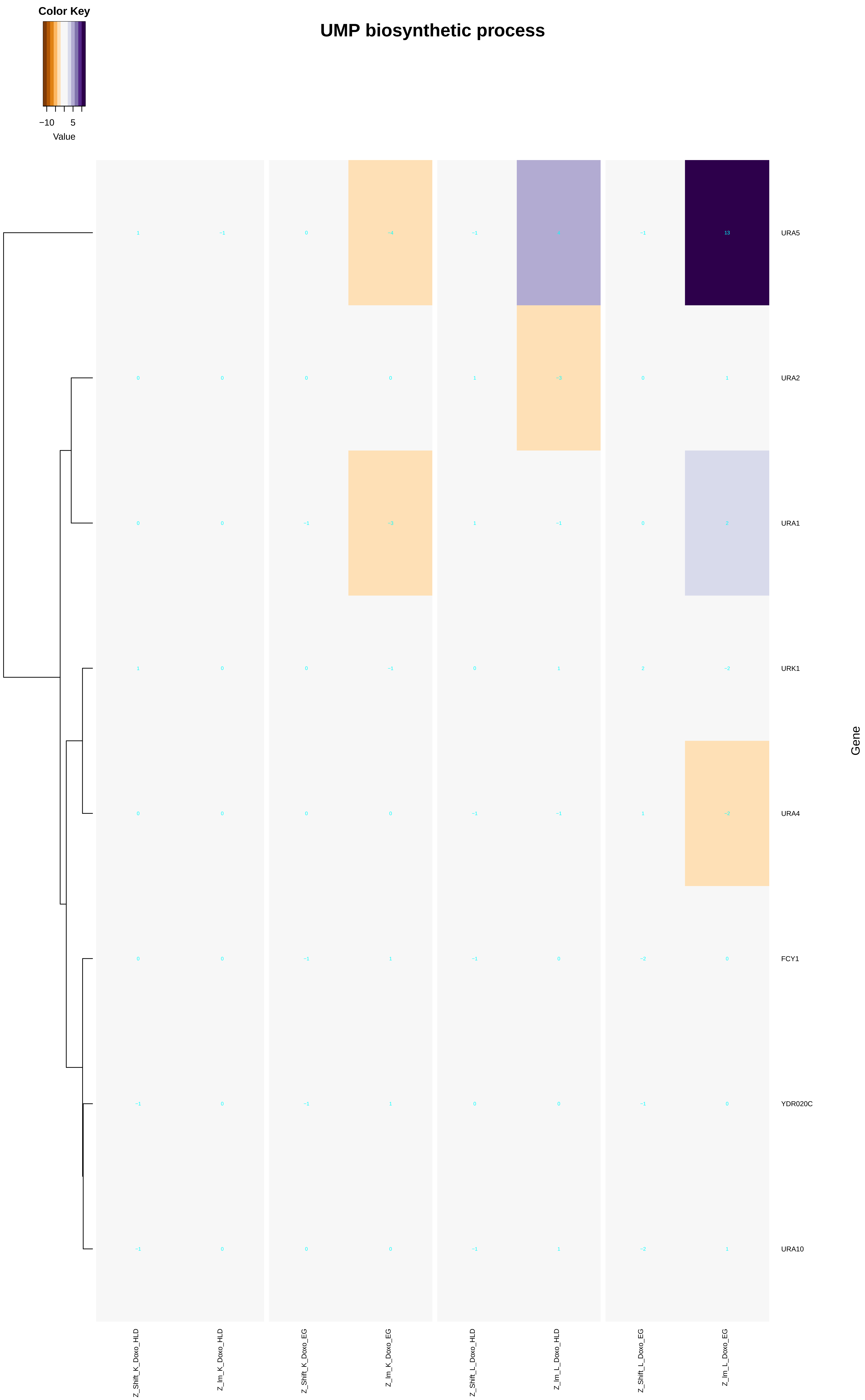
Color Key
−10
5
Value
UMP biosynthetic process
URA5
URA2
URA1
URK1
URA4
FCY1
YDR020C
URA10
Gene
Z_Shift_K_Doxo_HLD
Z_Im_K_Doxo_HLD
Z_Shift_K_Doxo_EG
Z_Im_K_Doxo_EG
Z_Shift_L_Doxo_HLD
Z_Im_L_Doxo_HLD
Z_Shift_L_Doxo_EG
Z_Im_L_Doxo_EG

CTP biosynthetic process

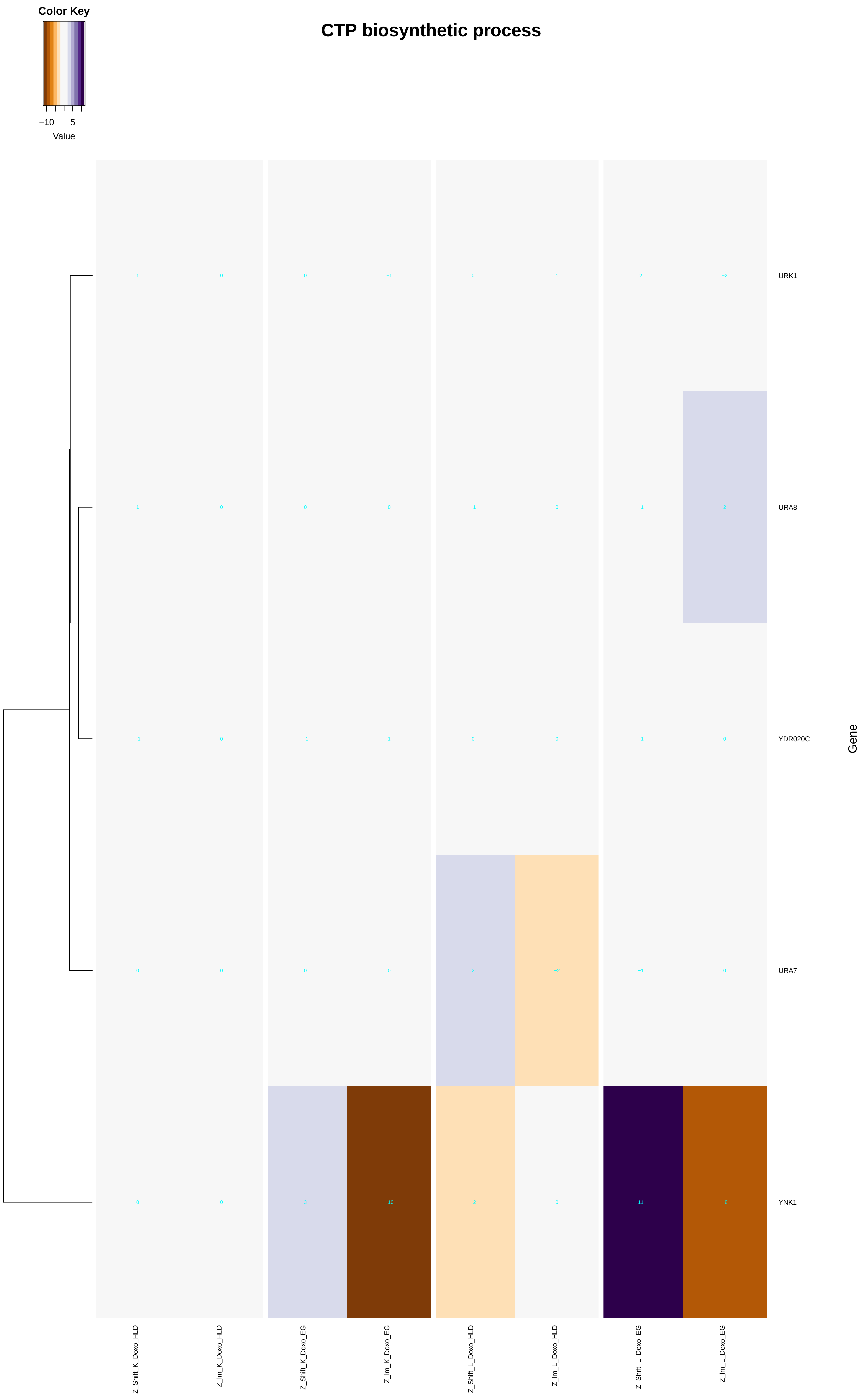

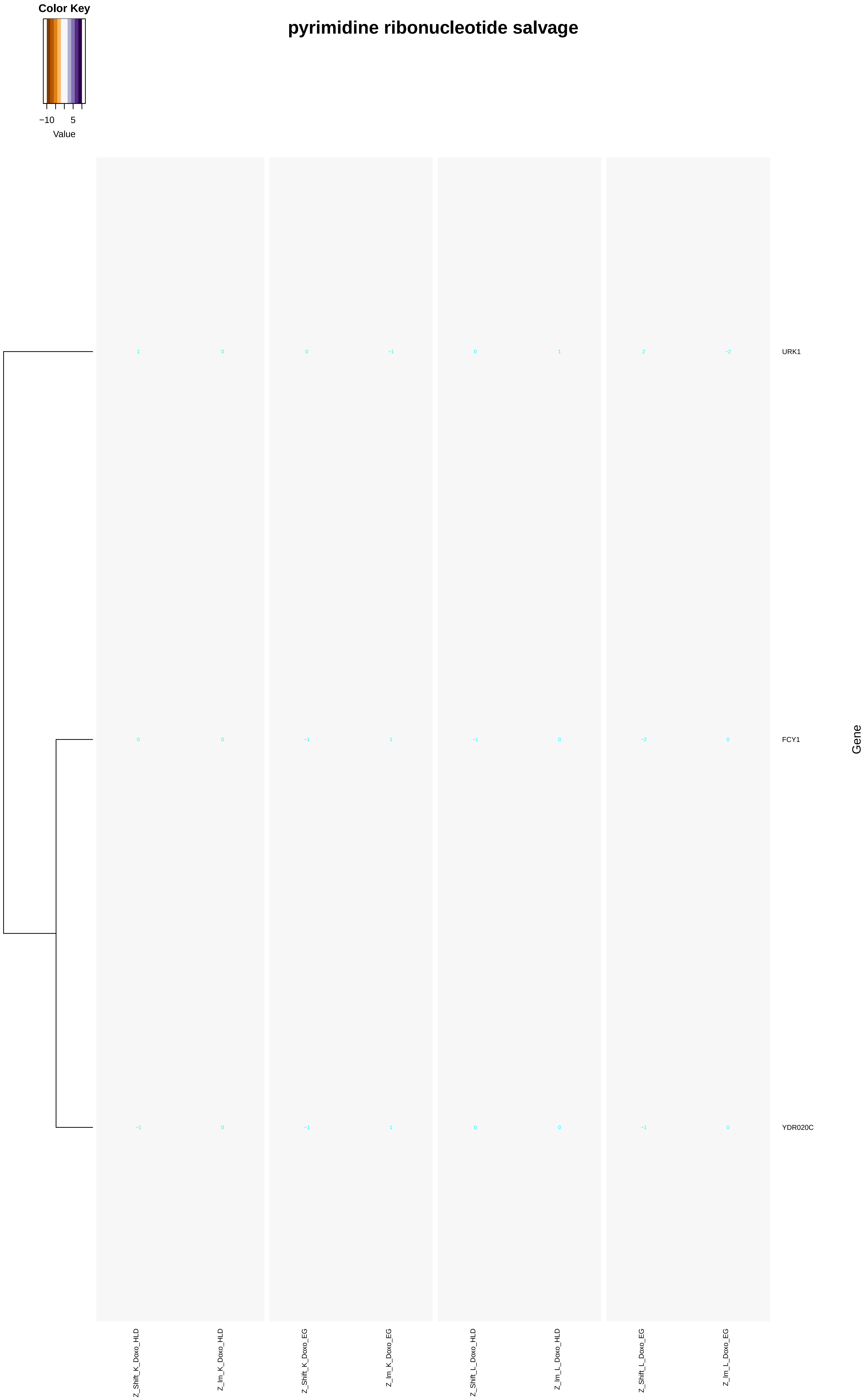
Color Key
−10
5
Value
pyrimidine ribonucleotide salvage
URK1
FCY1
YDR020C
Gene
Z_Shift_K_Doxo_HLD
Z_lm_K_Doxo_HLD
Z_Shift_K_Doxo_EG
Z_lm_K_Doxo_EG
Z_Shift_L_Doxo_HLD
Z_lm_L_Doxo_HLD
Z_Shift_L_Doxo_EG
Z_lm_L_Doxo_EG

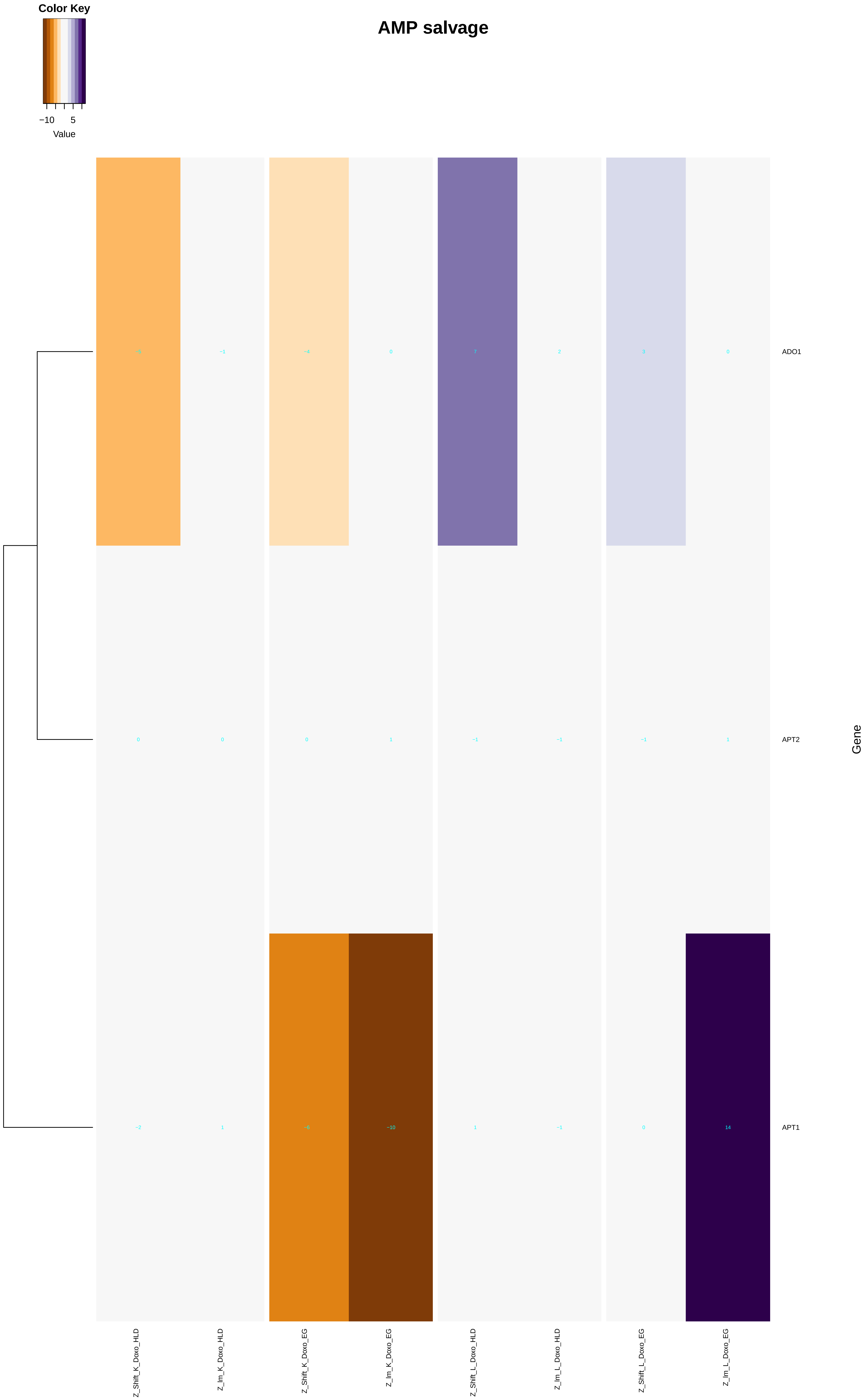

Color Key
−10
5
Value
AMP salvage
−5
−1
−4
0
7
2
3
0
ADO1
0
0
0
1
−1
−1
−1
1
APT2
−2
1
−6
−10
1
−1
0
14
APT1
Gene
Z_Shift_K_Doxo_HLD
Z_lm_K_Doxo_HLD
Z_Shift_K_Doxo_EG
Z_lm_K_Doxo_EG
Z_Shift_L_Doxo_HLD
Z_lm_L_Doxo_HLD
Z_Shift_L_Doxo_EG
Z_lm_L_Doxo_EG

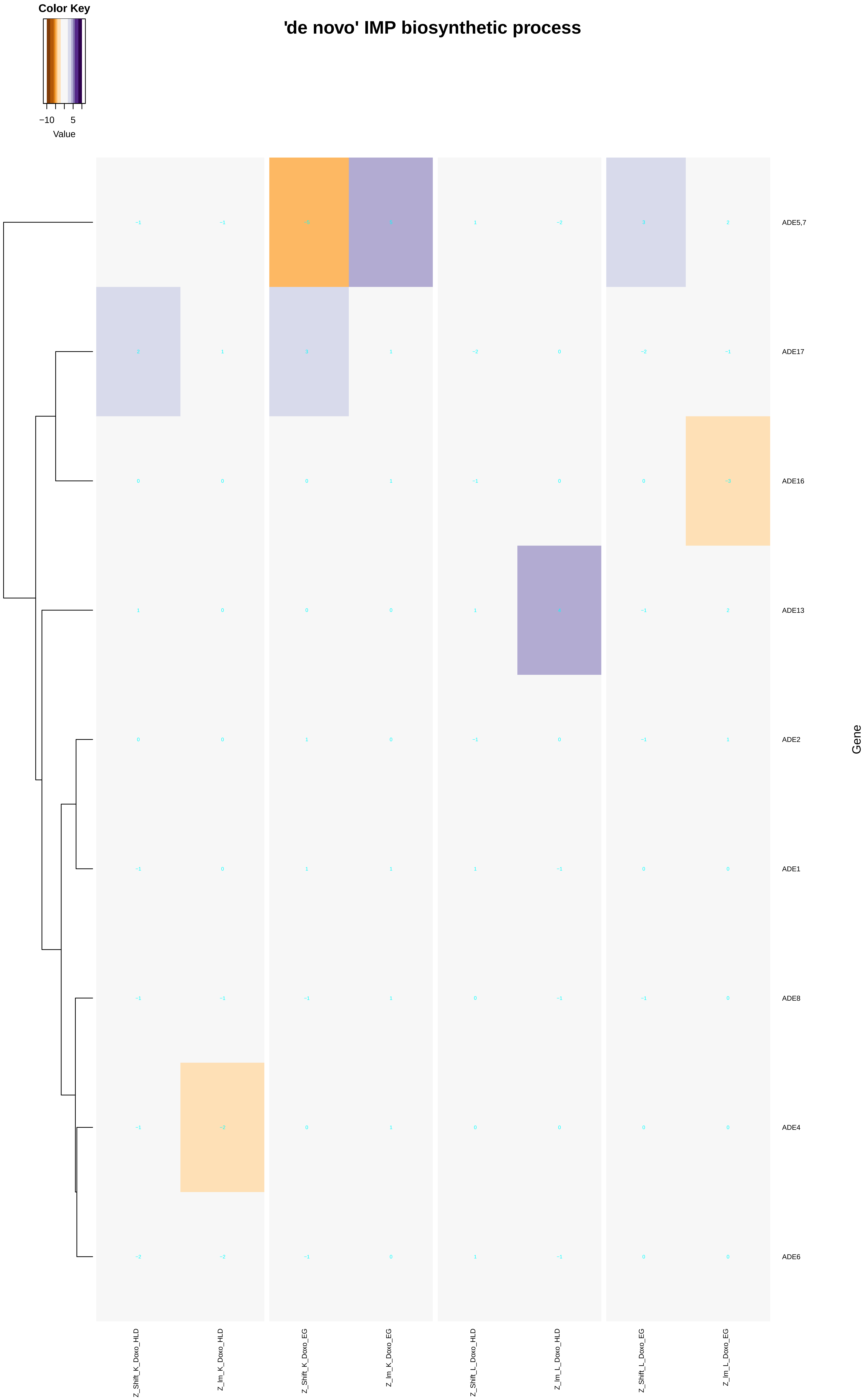
Color Key
−10
5
Value
'de novo' IMP biosynthetic process
ADE5,7
ADE17
ADE16
ADE13
ADE2
ADE1
ADE8
ADE4
ADE6
Gene
Z_Shift_K_Doxo_HLD
Z_Im_K_Doxo_HLD
Z_Shift_K_Doxo_EG
Z_Im_K_Doxo_EG
Z_Shift_L_Doxo_HLD
Z_Im_L_Doxo_HLD
Z_Shift_L_Doxo_EG
Z_Im_L_Doxo_EG

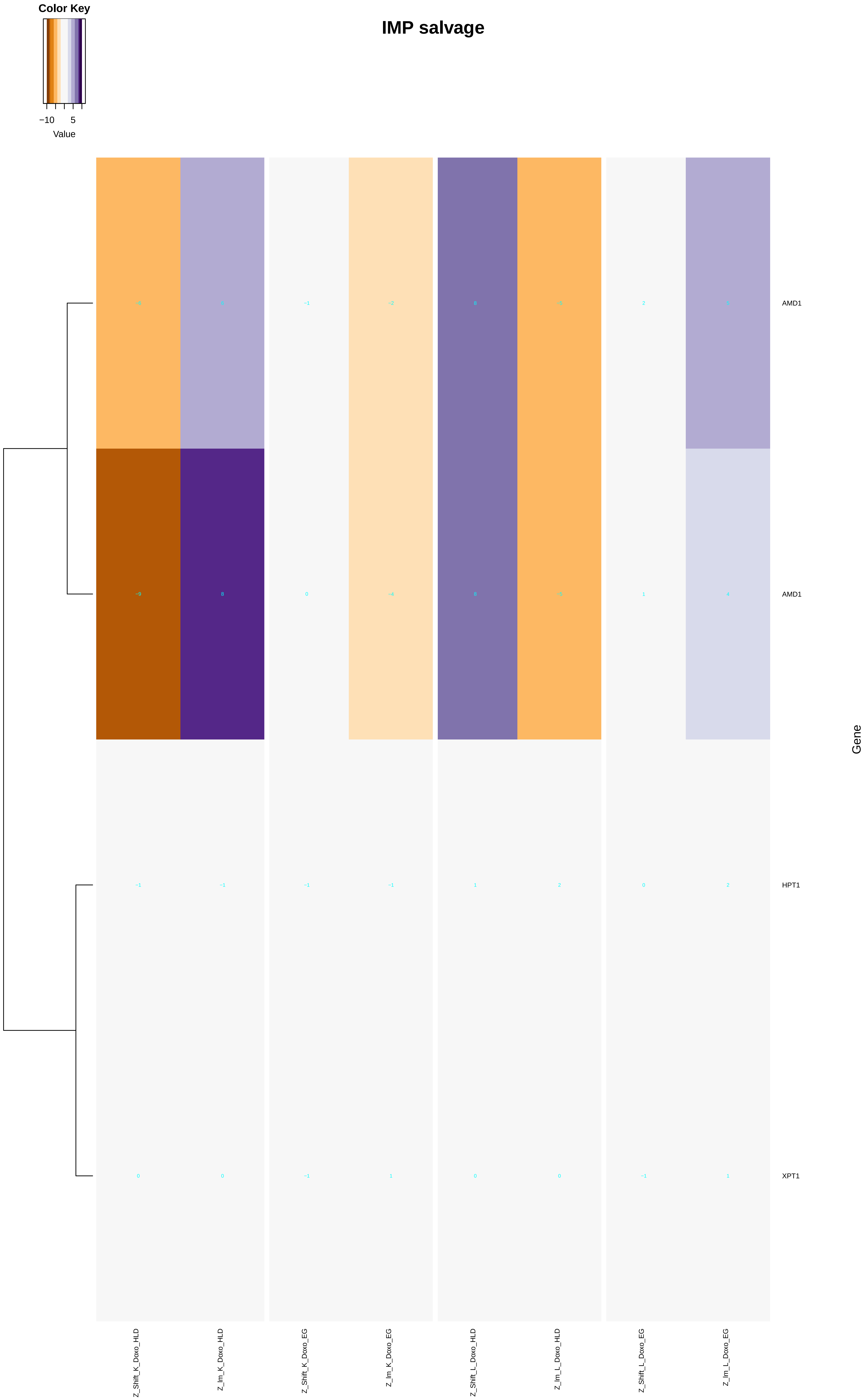
Color Key
−10
5
Value
IMP salvage
−6
6
−1
−2
8
−5
2
5
AMD1
−9
8
0
−4
8
−5
1
4
AMD1
−1
−1
−1
−1
1
2
0
2
HPT1
0
0
−1
1
0
0
−1
1
XPT1
Gene
Z_Shift_K_Doxo_HLD
Z_Im_K_Doxo_HLD
Z_Shift_K_Doxo_EG
Z_Im_K_Doxo_EG
Z_Shift_L_Doxo_HLD
Z_Im_L_Doxo_HLD
Z_Shift_L_Doxo_EG
Z_Im_L_Doxo_EG

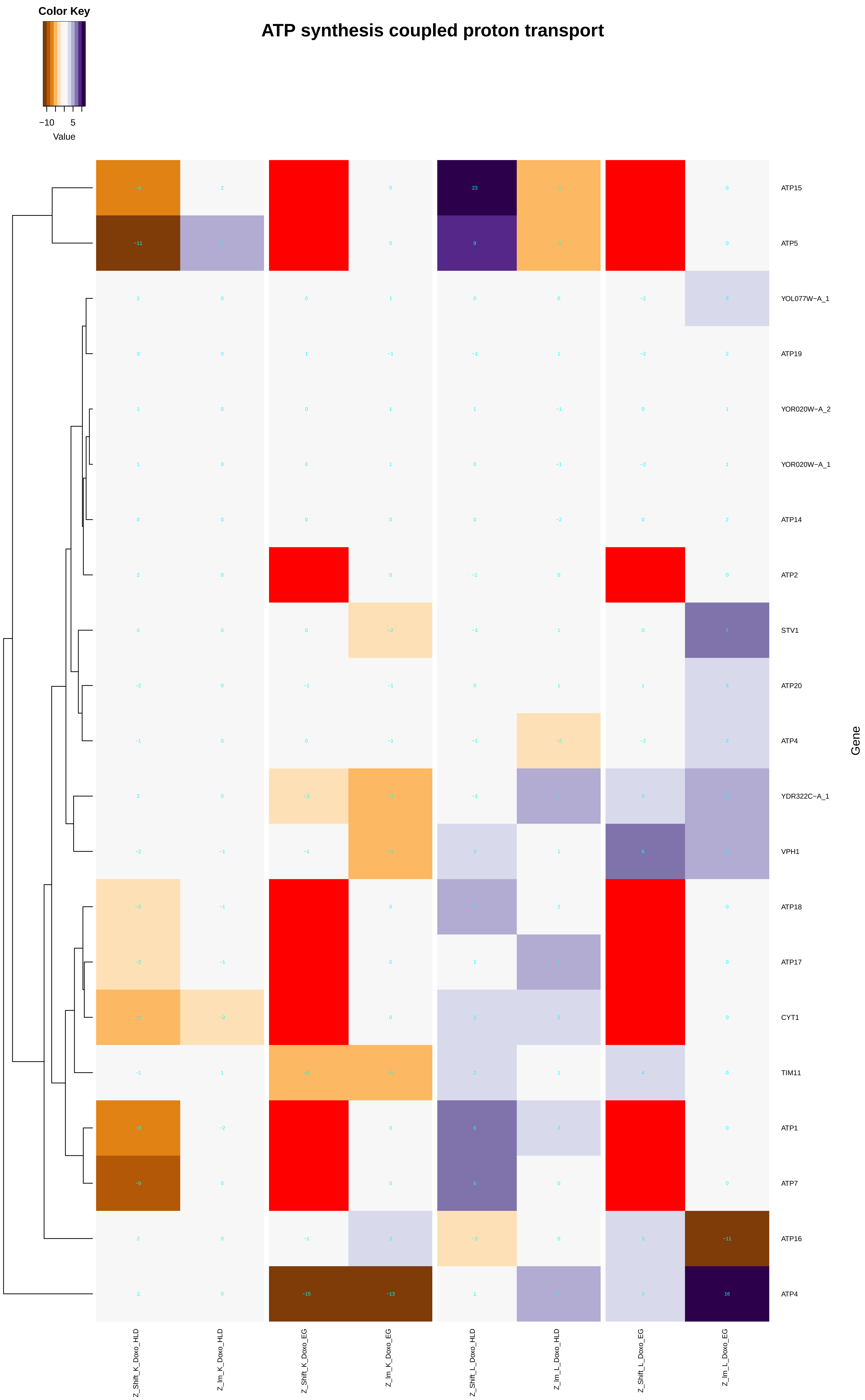
Color Key
−10
5
Value
ATP synthesis coupled proton transport
ATP15
ATP5
YOL077W−A_1
ATP19
YOR020W−A_2
YOR020W−A_1
ATP14
ATP2
STV1
ATP20
ATP4
YDR322C−A_1
VPH1
ATP18
ATP17
CYT1
TIM11
ATP1
ATP7
ATP16
ATP4
Gene
Z_Shift_K_Doxo_HLD
Z_Im_K_Doxo_HLD
Z_Shift_K_Doxo_EG
Z_Im_K_Doxo_EG
Z_Shift_L_Doxo_HLD
Z_Im_L_Doxo_HLD
Z_Shift_L_Doxo_EG
Z_Im_L_Doxo_EG

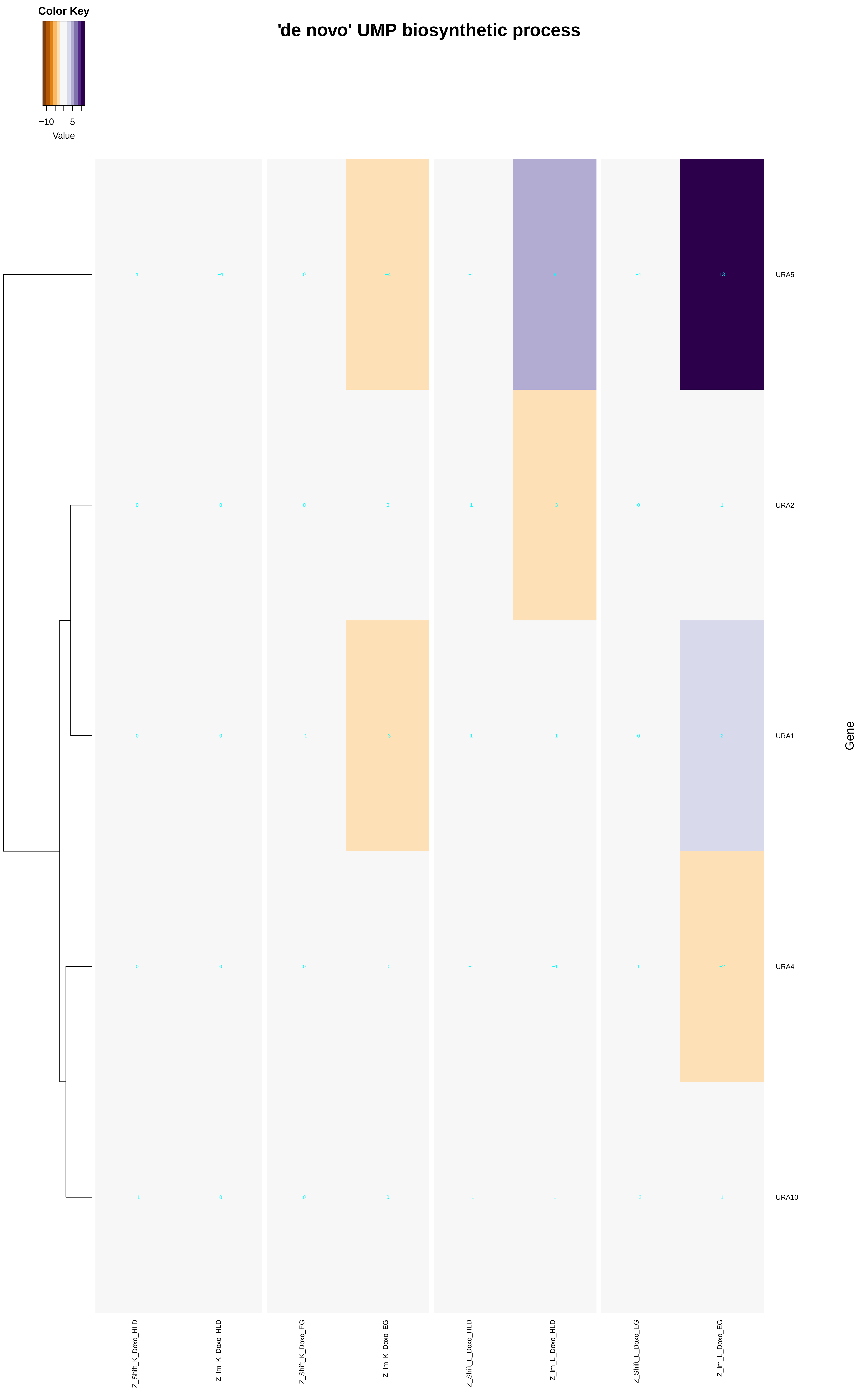

'de novo' UMP biosynthetic process
Color Key
−10
5
Value
URA5
URA2
URA1
URA4
URA10
Gene
Z_Shift_K_Doxo_HLD
Z_lm_K_Doxo_HLD
Z_Shift_K_Doxo_EG
Z_lm_K_Doxo_EG
Z_Shift_L_Doxo_HLD
Z_lm_L_Doxo_HLD
Z_Shift_L_Doxo_EG
Z_lm_L_Doxo_EG

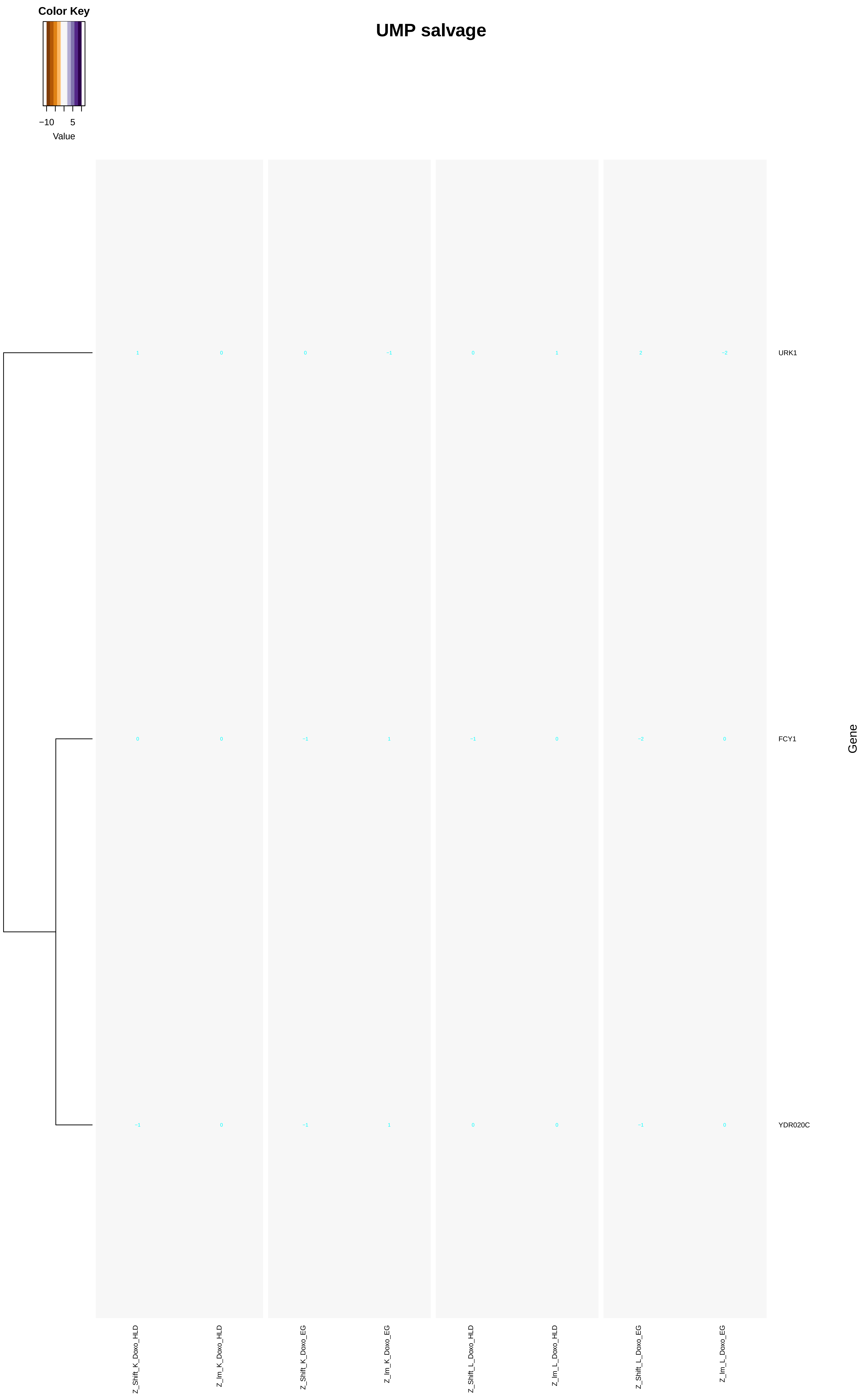
Color Key
−10
5
Value
UMP salvage
URK1
FCY1
YDR020C
Gene
Z_Shift_K_Doxo_HLD
Z_Im_K_Doxo_HLD
Z_Shift_K_Doxo_EG
Z_Im_K_Doxo_EG
Z_Shift_L_Doxo_HLD
Z_Im_L_Doxo_HLD
Z_Shift_L_Doxo_EG
Z_Im_L_Doxo_EG
